# Maternal and child predictors of vascular health in infancy and early childhood

**DOI:** 10.1038/s41390-025-04260-1

**Published:** 2025-10-20

**Authors:** Reeja F. Nasir, Nathalie Kizirian, Ravin Lal, Angelika Zankl, Allison Grech, Marjan M. Haghighi, Michael R. Skilton, Adrienne Gordon

**Affiliations:** 1 The Boden Initiative, Charles Perkins Centre, University of Sydney, Camperdown, NSW Australia; 2 Faculty of Medicine and Health, University of Sydney, Camperdown, NSW Australia; 3 Charles Perkins Centre, University of Sydney, Camperdown, NSW Australia; 4 Sydney Institute for Women, Children and their Families, Sydney Local Health District, Sydney, NSW Australia; 5 Department of Obstetrics, Gynaecology and Neonatology, Royal Prince Alfred Hospital, Sydney Local Health District, Sydney, NSW Australia

## Abstract

**Background:**

Excessive weight gain (EWG) in childhood may increase cardiovascular disease risk in adulthood, but its relationship to early vascular health remains poorly understood. This study investigated the relationship between EWG and aortic intima-media thickness (aIMT), a non-invasive ultrasound imaging marker of early vascular health, and explored maternal and child factors that may influence growth and aIMT during early childhood.

**Methods:**

Mother–child pairs from the BABY1000 pilot cohort were followed from early pregnancy to 24 months. Maternal and birth data were collected via questionnaires and medical records. Child anthropometry and aIMT were measured at 6 weeks and 24 months. EWG was defined as a > 0.67 SD increase in weight-for-length/height z-score from birth. Maternal pregnancy diet and child diet at 24-months was assessed with age-specific Australian Eating Survey® food frequency questionnaires. Linear regression was used to examine associations among growth, diet, and aIMT.

**Results:**

Of 182 births, 102 infants at 6 weeks and 83 children at 24 months had aIMT data. 26% of children at 6 weeks and 45% at 24 months had EWG, but it was not associated with aIMT at either follow-up. No associations were found between maternal or child diet and growth or aIMT.

**Conclusions:**

Measuring aIMT in early childhood is feasible; however, larger studies are needed to clarify early-life influences on cardiovascular health.

**Impact:**

- Excessive weight gain (EWG) in childhood is associated with increased risk for later-life cardiovascular disease.
- Understanding these associations is complicated by confounding from maternal and perinatal factors and the long interval between exposure and disease outcomes.
- In this pilot study, we longitudinally measured aortic intima-media thickness (aIMT) in children, an age-appropriate marker of vascular health, and examined associations with early growth, maternal, and perinatal factors.
- EWG was not associated with aIMT during early childhood.
- Early childhood is important for long-term health promotion; however, more research is needed to clarify how exposures during this period affect later cardiovascular health.

## Introduction

The Developmental Origins of Health and Disease (DOHaD) hypothesis proposes that adverse early-life exposures, particularly during the prenatal period, can promote structural, physiological, and metabolic changes within an individual. These adaptations can subsequently interact unfavourably with the postnatal environment, increasing the risk for later-life cardiometabolic disease.^1,2^ Consistent with this hypothesis, epidemiological studies have found that excessive weight gain (EWG) in infancy, especially following a period of growth restriction in utero, is associated with a higher incidence of cardiovascular disease (CVD)-related events in adulthood.^3^ Optimising nutrition during this critical period, including maternal diet during pregnancy, duration of exclusive breastfeeding, and child diet, has been suggested as a potential modifiable factor in reducing these risks.^4^

One potential mechanism linking EWG to increased CVD risk involves changes in arterial vascular health. Indeed, retrospective studies have shown that EWG is associated with thicker and stiffer arterial walls in childhood, adolescence, and adulthood.^5–9^ However, the delay between the exposure early in life and the measurement of vascular health later in life can introduce significant confounding. Longitudinal studies with repeated measures of age-appropriate vascular markers are necessary to better characterise how early growth patterns, and any effects of nutrition, influence cardiovascular health trajectories over time.

To that end, our previous work has identified aortic intima-media thickness (aIMT), a surrogate marker for pre-clinical atherosclerosis, as an age-appropriate measure for exploring associations between putative risk factors and vascular health in pre-pubertal children.^10^ We, and others, have also shown it to be feasible to perform in infants.^11,12^ Previous studies have explored the link between EWG during infancy and aIMT in pre-selected exposure-specific groups,^13^ reducing the generalisability of their findings. To date, only one study^13^ has evaluated the above prospectively in an unselected birth cohort and during early childhood. In their cohort of 835 mother-infant pairs, McCloskey et al.^13^ found that higher birthweight, birth adiposity, and greater weight and fat mass gain from birth to 6 weeks, independent of birthweight, were associated with increased aIMT at 6 weeks. However, the study lacked longitudinal measures to determine whether these associations persisted into later infancy.

Building on this, the primary aim of this study was to investigate whether EWG during infancy (6 weeks) and early childhood (24 months) was associated with aIMT. This was a pre-specified secondary analysis of the BABY1000 pilot study, a prospective birth cohort study based in Sydney, Australia, which aimed to identify modifiable exposures before and during pregnancy that may impact the health of both mother and child. We hypothesised that children with EWG during these periods would have increased aIMT at follow-up compared to those who did not significantly deviate from their growth trajectory, as determined by size at birth. Furthermore, we anticipated this effect would be most pronounced in those born small-for-gestational age (SGA), a proxy for growth restriction. In addition to our primary aim, we explored whether maternal characteristics and diet during pregnancy, as well as child birth characteristics and diet, were associated with growth velocity and aIMT, to identify potential modifiable exposures in the perinatal period that may influence early cardiovascular health.

## Methods

### Participant recruitment

**Fig. 1:**
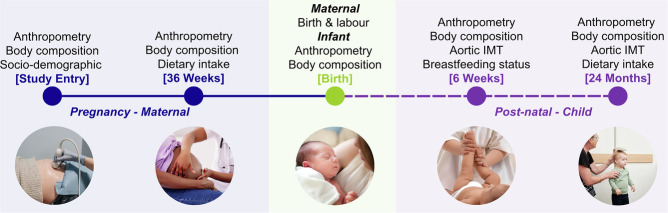
Timeline of data collection and summary of measurements collected at each timepoint for the BABY1000 pilot study included in this sub-analysis. IMT intima-media thickness.

A detailed description of the BABY1000 pilot study design, recruitment, inclusion and exclusion criteria, data collection timeline, and methodology has been outlined in a previous cohort description.^14^ Briefly, women older than > 18 years of age were recruited at either pre-conception or at gestation less than 13 weeks and 0 days. Recruitment was conducted between December 2017 and August 2020 at pre-conception and antenatal clinics within the RPAH Foetal Medicine Unit at Royal Prince Alfred Hospital (RPAH) Women’s and Babies and the Charles Perkins Centre (CPC) RPA Clinic. Follow-up was conducted throughout pregnancy and continued until the child’s second birthday. As most participants were recruited at gestation less than 13 weeks and 0 days, this was defined as “study entry”. For the few mothers who had been recruited at pre-conception and were active at birth, data from their 12-week visit was used as the study entry point. Data from study entry, 36 weeks’ gestation, birth, 6 weeks, and 24 months follow-up is included in this analysis (Fig. 1).

An overarching aim of the BABY1000 pilot was to determine the feasibility and acceptability of embedding longitudinal research within routine clinical care. As such, the selection of follow-up timepoints was informed by existing maternal and infant postnatal care schedules and the public health context at the time. The 6-week timepoint aligned with a routine maternal postnatal visit, offering a pragmatic and low-burden opportunity to assess the infant. The 24-month timepoint was adopted in response to COVID-19 pandemic-related disruptions, which led to the early discontinuation of the planned 12-month visit. 

### Maternal demographics, anthropometry, body composition, labour, and birth details

Height was measured at study entry without shoes to the nearest 0.1 cm using a wall-mounted stadiometer (HR-200 Wall-mounted Height Rod, Tanita Australia, Kewdale, WA, Australia; or similar). Maternal weight was measured using a digital scale without shoes and light clothing to the nearest 0.1 kg (BC-418 Segmental Body Composition Analyzer, Tanita Australia; or similar). Body Mass Index (BMI) was calculated as kg/m^2^. Body composition was measured by air-displacement plethysmography (BOD POD, COSMED Inc., Chicago, IL) as per the manufacturer’s protocol at study entry and 36 weeks’ gestation. Air-displacement plethysmography measures whole-body volume, which is then used to calculate fat mass and fat-free mass,^15^ and has been used in previously in pregnancy.^16^ Maternal demographics such as ethnicity, education, and occupation were recorded via a self-reported study entry questionnaire. Pregnancy and birth details were collected from medical records. Diabetes in pregnancy included both gestational diabetes (diabetes first diagnosed during pregnancy) and pre-existing diabetes. Hypertensive disorders in pregnancy were defined as chronic hypertension, gestational hypertension, pre-eclampsia/eclampsia and chronic hypertension superimposed with pre-eclampsia/eclampsia. Definitions were consistent with the NSW Perinatal Data Collection data dictionary.^17^ Gestational weight gain was defined as the change in weight from study entry to 36 weeks’ gestation. Gestational age was calculated from the first-trimester ultrasound. Infant size for gestational age (small, large, and appropriate) was calculated using the Fenton 2013 Growth Charts.^18^

### 6-week infant anthropometry, body composition, and aortic intima-media thickness

Study appointments at 6 weeks of age were conducted in a quiet, temperature-controlled examination room at the Neonatal Intensive Care Unit (NICU) at the RPAH. The pre-specified acceptable age range for this visit was 6 weeks ±1 week; however, there was frequent re-scheduling due to the COVID-19 pandemic restrictions. Exclusion of individuals who exceeded this age range did not materially affect our findings and, as such, they were included in our final analysis. Anthropometric measurements were taken with the infant unclothed and without a diaper. Body weight was measured using a standard newborn scale (Tanita Australia). Length was measured in the supine position using an infant length board (Easy-Glide Bearing Infantometer; Perspective Enterprises, Portage, MI). Body composition was measured using air-displacement plethysmography (PEA POD, COSMED Inc.). The PEA POD is specifically designed for infants up to six months of age.^19,20^ Where possible, the measurement of aIMT aligned with published best-practice guidelines.^10,21^ Prior to the measurement, the infant was settled and placed in a supine position. Then, a longitudinal straight non-branched segment of the abdominal aorta between the umbilicus and xiphisternum was imaged using high-resolution B-mode ultrasonography (EPIQ 5; Philips Medical Systems, Best, NB, Netherlands) with a high-frequency linear array probe (18–5 MHz). Two neonatologists and experienced sonographers acquired the images (A.G. and A.Z.). Far-wall IMT was measured offline using a validated semi-automated edge-detection software (Carotid Analyzer, Version 5; Medical Imaging Applications, Coralville, IA) by an experienced observer (R.N.), with manual tracing adjustment when required. Maximum aIMT and minimum vessel diameter were averaged across three cardiac cycles at end-diastole. Maximum aIMT was our primary outcome, given the focal nature of atherosclerosis.^10^

### 24-month maternal anthropometry; child anthropometry, body composition, and aortic intima-media thickness

Study appointments at 24 months were conducted in a quiet, temperature-controlled examination room in a clinical research facility. The pre-specified acceptable age range for this visit was 24–30 months. A broader age range was specified to accommodate working parents. Exclusion of individuals who exceeded this age range did not materially affect our findings and, as such, they were included in our final analysis. For both mother and child, body weight was measured using a digital scale without shoes and light clothing (BC-418 Segmental Body Composition Analyzer). Standing height was measured without shoes using a wall-mounted stadiometer (HR-200 Wall-mounted Height Rod). Child body composition was measured by air-displacement plethysmography. The BOD POD has a paediatric option for children aged 2–6 years whereby a small seat is added to the machine and calibrated.^22^ A minimum of three sequential measurements were taken, and child status was recorded during the measurement, i.e., calm or crying. aIMT was collected and analysed per the 6-week visit, except that an updated ultrasound machine and a 12-3 MHz linear-array probe (EPIQ 7; Philips Medical Systems) were used for image acquisition. Children were kept supine for the measurement and were settled using distractions such as toys, stickers, or a screen.^21^ The reliability of the aIMT measurement was assessed by retesting 15 randomly selected scans at least one month apart by the same observer; the intraclass-correlation coefficient for maximum IMT was 0.86 (95% CI 0.63, 0.95), indicating “good” reliability.^23^

### Maternal and child diet

Maternal diet during pregnancy and child diet at 24 months were assessed using the Australian Eating Survey® Food Frequency Questionnaire (AES FFQ), which includes contemporary foods and drinks typically consumed in Australia. The online self-administered questionnaire is designed to measure dietary intake over the previous 3–6 months. A validated adult version of the AES FFQ was completed by mothers at 36 weeks’ gestation to capture habitual intake during pregnancy,^24^ while a separate, age-appropriate version validated for Australian toddlers was used to assess child diet at 24 months.^25^ The child’s dietary assessment was completed by the parent primarily responsible for their diet (the biological mother in almost all cases) at the 24-month follow-up. A detailed description of maternal diet and its quality during early and late pregnancy has been reported previously in a subset of participants.^26^ This paper reports data only from those with a paired aIMT measurement.

### Definition of growth velocity and excessive weight gain

Z-scores were calculated using the World Health Organisation (WHO) Child Growth Standards as recommended by the Australian National Health and Medical Research Council (NHMRC) Dietary Guidelines.^27^ Growth velocity was defined as the difference in weight-for-length/height z-score across two distinct intervals: birth to 6 weeks (early infancy) and birth to 24 months (later infancy to early childhood).^28,29^ Using weight-for-length z-scores, rather than weight z-scores alone, allows us to account for weight gain attributable to simultaneous increases in length/height. EWG was defined separately for each interval as a positive change of more than 0.67 standard deviations (SD) in weight-for-length/height z-score, consistent with established criteria for clinically meaningful rapid growth.^28,29^ A decrease greater than 0.67 SD was classified as “decelerated growth,” while a change within ±0.67 SD was considered “on-track.” Z-scores were calculated using actual age at presentation to account for variability in visit timing.

### Statistical analysis

Descriptive data were presented as means (SDs) for continuous variables and n (%) for categorical variables. Data was visually assessed for normality distribution and confirmed using the Kolmogorov-Smirnov test. Independent sample *t* tests with equal variance assumed were used to calculate *p*-values for continuous variables such as maternal age and BMI. Pearson chi-squared test was used to compare categorical variables such as maternal ethnicity and education.

Regression analyses were conducted to reflect the study’s primary and secondary aims. The primary analysis focused on the association between EWG and aIMT measured at the corresponding timepoints. Secondary exploratory analyses examined associations between: (1) maternal characteristics and pregnancy total energy and macronutrient intake (as % total daily energy) with infant outcomes at birth and 6 weeks; (2) infant birth characteristics with growth and aIMT at 6 weeks and 24 months; and (3) exclusive breastfeeding at 6 weeks and child total energy and macronutrient intake (as % total daily energy) at 24 months with growth and aIMT. This approach allowed us to evaluate potentially modifiable maternal and child exposures across the perinatal and early childhood period.

For multivariable regression, we considered variables that are clinically important (e.g., maternal weight), variables that have been previously shown to differentially affect arterial vasculature (e.g., infant sex), as well as variables with a 2-sided *p* value < 0.05 in univariate regression. Statistical significance was inferred as a 2-sided *p* value < 0.05 for all tests. Statistical analyses were performed using SPSS (Version 26.0; IBM Corporation, Armonk, NY) and figures were created using GraphPad Prism (Version 8; GraphPad Software, Inc., San Diego, CA) and Microsoft PowerPoint (Version 2506; Microsoft Corporation, Redmond, WA).

The study was conducted in accordance with the Declaration of Helsinki and the NHMRC Statement on Ethical Conduct in Research Involving Humans^30^ and approved by the Human Research Ethics Committee of the Sydney Local Health District (Protocol No. X17-0019). Participation was voluntary, and informed consent was obtained from each participant’s parent or guardian.

## Results

### Participant characteristics

#### Maternal characteristics

**Fig. 2:**
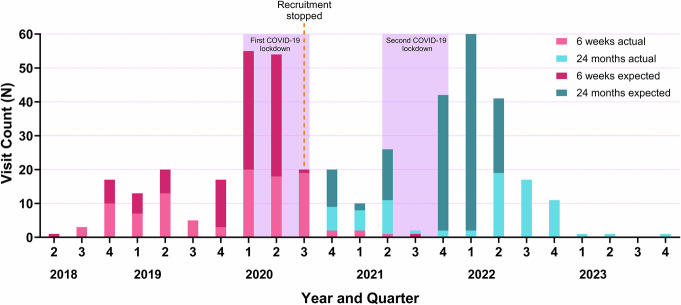
Impact of the COVID-19 pandemic on participant attendance at post-birth follow-up visits in the BABY1000 pilot study. Bar chart illustrating expected versus actual visit attendance at the 6-week and 24-month follow-ups, in relation to the timing of the COVID-19 pandemic lockdowns in Sydney, Australia.

**Fig. 3:**
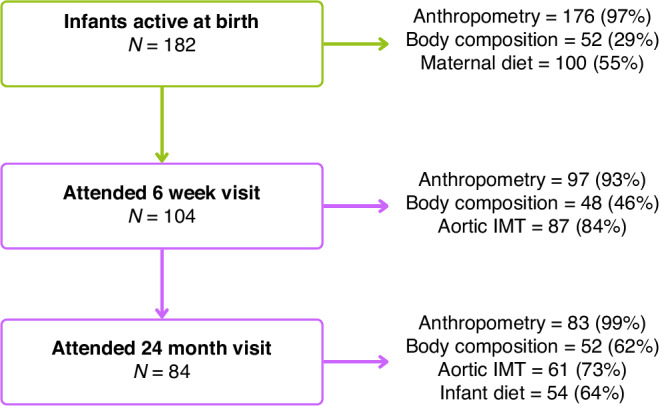
Summary of participant retention and data availability. Flowchart depicting number of participants active at birth, attendance at each follow-up, and completion rates [n (%)] for key child assessments in the BABY1000 pilot study.

Maternal and child characteristics at baseline and those who attended the 6-week and 24-month follow-up are summarised in Tables 1,  2, respectively. Figure 2 illustrates the impact of the COVID-19 pandemic on participant attendance at follow-up visits after birth. Figure 3 summarises participant retention and data completeness for key child outcomes at each timepoint. Mothers who participated in the BABY1000 study had a mean age of 33.3 (SD 4.3), a BMI of 24.9 (4.4) kg/m^2^, and gained, on average, 10.7 (4.1) kgs during pregnancy. Most participants were primiparous (58.7%), identified as Caucasian (53.8%), and were tertiary educated (66.2%). The cohort was similar to birthing women at RPAH except BABY1000 participants were more likely to be primiparous and tertiary educated.^31^ Maternal body fat percentage was significantly lower in those who attended the 24-month visit (*p* = 0.02). Although statistically significant this finding may not be clinically relevant due to the small number of women (*N* = 24) with a body composition measurement at study entry who attended the 24-month follow-up.

**Table 1:** Maternal characteristics at baseline and for those who attended the 6-week and 24-month follow-up of the BABY1000 pilot study.

*Characteristic*	Baseline (*N* = 225) N (%)	6-week follow-up (*N* = 102) N (%)	6-week vs. Baseline *p value*	24-month follow-up (*N* = 83) N (%)	24-month vs. Baseline *p value*
Age at presentation (years)					
Mean (SD)	33.3 (4.2)	33.6 (4.3)	0.31	33.9 (3.5)	0.10
20–29	32 (14.2)	11 (10.7)		6 (7.2)	
30–34	115 (51.1)	53 (52.4)		49 (59.0)	
35–39	63 (28.0)	30 (29.1)		21 (25.3)	
≥40	15 (6.7)	8 (7.8)		7 (8.4)	
BMI (kg/m^2^)					
Mean (SD)	24.9 (4.4)	25.3 (4.5)	0.22	24.9 (4.3)	0.99
<18.5	5 (2.2)	3 (2.9)		3 (3.6)	
18.5–24.9	119 (52.9)	50 (49.0)		41 (49.4)	
25–29.9	67 (29.8)	30 (29.4)		29 (34.9)	
≥30	24 (10.7)	15 (14.7)		8 (9.6)	
Missing BMI data	10 (4.4)	3 (3.9)		2 (2.4)	
Weight (kg), mean (SD)	67.3 (13.4)	69.4 (14.0)	0.06	67.9 (13.4)	0.66
Height (cm), mean (SD)	164.0 (7.6)	164.9 (7.6)	0.07	164.7 (7.8)	0.29
Fat mass (%), mean (SD)	30.9 (7.7)	31.0 (7.2)	0.98	28.4 (7.1)	0.02*
Ethnicity					
Caucasian	121 (53.8)	63 (61.8)	0.13	49 (59.0)	0.12
Asian	44 (19.6)	19 (18.6)		16 (19.3)	
South Asian	25 (11.0)	6 (5.9)		6 (7.2)	
Pacific Islander	4 (1.8)	3 (2.9)		0 (0)	
Middle Eastern	5 (2.2)	2 (2.0)		1 (1.2)	
Other	15 (6.6)	9 (8.8)		9 (10.8)	
Missing ethnicity data	11 (4.9)	0 (0)		2 (2.4)	
Education					
Less than high school	2 (0.9)	1 (1.0)	0.54	0 (0)	0.21
High school	5 (2.2)	4 (3.9)		1 (1.2)	
More than high school	149 (66.2)	82 (80.4)		70 (84.3)	
Missing education data	69 (30.7)	15 (14.7)		12 (14.5)	
Previous live births					
0	132 (58.7)	60 (58.8)	0.63	47 (56.6)	0.71
1	78 (34.7)	34 (33.3)		29 (34.9)	
2	11 (4.9)	7 (6.9)		6 (7.2)	
≥3	4 (1.7)	1 (1.0)		1 (1.2)	
Diabetes in pregnancy^a^	35 (15.6)	19 (18.6)	0.70	17 (20.5)	0.37
Hypertensive disorders in pregnancy^b^	6 (2.7)	4 (3.9)	0.47	3 (3.6)	1.00

Values are mean (SD) for continuous variables and n (%) for categorical variables.

Independent sample t-test and chi-squared test were used to compare characteristics between baseline and follow-up for continuous (e.g., age and BMI) and categorical (e.g., ethnicity and education) variables, respectively.

Bold values indicate significant differences between groups (*p* < 0.05).

^a^Diabetes in pregnancy was defined as both gestational diabetes (diabetes first diagnosed during pregnancy) and pre-existing diabetes.

^b^Hypertensive disorders in pregnancy were defined as chronic hypertension, gestational hypertension, pre-eclampsia/eclampsia and chronic hypertension superimposed with pre-eclampsia/eclampsia.

*BMI* body mass index.

**Table 2:** Child characteristics at birth and for those who attended the 6-week and 24-month follow-up of the BABY1000 pilot study.

*Characteristic*	Baseline (*N* = 208) N (%)	6-week follow-up (*N* = 104) N (%)	6-week *vs*. Baseline *p value*	24-month follow-up (*N* = 84) N (%)	24-month vs. Baseline *p value*
Sex					
Female	108 (51.9)	50 (48.1)	0.27	40 (47.6)	0.31
Male	100 (48.1)	54 (51.9)		44 (52.4)	
Gestational age (weeks)					
Mean (SD)	39.3 (1.5)	39.4 (1.3)	0.33	39.3 (1.3)	0.99
< 37	14 (6.7)	5 (4.8)		5 (6.0)	
37–41	194 (93.3)	99 (95.2)		79 (94.0)	
Birthweight (g)					
Mean (SD)	3360 (497)	3403 (473)	0.21	3363 (441)	0.94
< 2499	11 (5.3)	5 (4.8)		3 (3.6)	
2500–2999	36 (17.3)	14 (13.5)		17 (20.2)	
3000–3999	144 (69.2)	73 (70.2)		57 (67.9)	
≥ 4000	17 (8.2)	12 (11.5)		7 (8.3)	
Length (cm), mean (SD)	50.2 (2.9)	50.3 (3.5)	0.25	50.2 (2.1)	0.56
Birthweight centile					
AGA	186 (89.4)	92 (88.5)	0.58	75 (89.3)	0.33
SGA	13 (6.3)	6 (5.8)		7 (8.3)	
LGA	9 (4.3)	6 (5.8)		2 (2.4)	
Head circumference (cm), mean (SD)	34.3 (1.5)	34.5 (1.4)	0.40	34.5 (1.4)	0.75
Fat mass (%), mean (SD)	11.5 (3.3)	11.4 (3.3)	0.94	11.5 (3.0)	0.91
Admission to neonatal intensive care	26 (12.5)	14 (13.5)	0.34	7 (8.3)	0.61

Values are mean (SD) for continuous variables and n (%) for categorical variables.

Independent sample *t* test and chi-squared test were used to compare characteristics between baseline and follow-up for continuous (e.g., birthweight) and categorical (e.g., sex) variables, respectively.

*AGA* appropriate for gestational age, *LGA* large for gestational age, *SGA* small for gestational age.

#### Child characteristics

The average gestational age at birth was 39.3 (1.5) weeks, birthweight was 3360 (497) grams, and 52% of infants were female. Less than 10% of infants were born SGA (*N* = 13, 6.3%), which is lower than the expected proportion for the Australian population, indicating a generally healthy study population. The mean age at the 6-week follow-up (*N* = 102) was 67.6 (37.3) days, weight was 5.3 (0.9) kg, and recumbent length was 57.9 (3.2) cm. Of those who attended the 6-week visit, most infants were still exclusively breastfed (*N* = 52, 52.5%). Males and females were of comparable age, but males tended to be larger, longer and gained more weight than females (*p* < 0.05). The mean age at the 24-month follow-up (*N* = 83) was 2.3 (0.2) years, weight was 13.7 (1.6) kg, and standing height was 91.5 (4.4) cm. Males and females were comparable. Children lost to follow-up did not differ significantly from the study population with respect to birth characteristics (Table 2).

#### Child aortic-intima media thickness at 6-week and 24-month follow-up

Image acquisition rates for aIMT were significantly higher at the 6-week follow-up (100%) compared to the 24-month follow-up (88.1%), as infants were easier to settle. The proportion of IMT scans of sufficient quality for analysis, after removal of low-quality images, showed a similar trend (82.7% *vs*. 72.6%). Although our overall rate of missing aIMT data exceeded the recommended 10% threshold,^10^ it was comparable to other studies in similarly aged children.^12^ Maximum aIMT at 6 weeks was 602 (62) µm, and the vessel diameter was 5846 (603) µm. These values differed from those reported in the Barwon Infant Study, another unselected Australian birth cohort which also used automated edge-detection software, where the mean maximum aIMT was 719 (67) µm and vessel diameter was 4980 (535) µm.^13^ Maximum aIMT at 24-months was 668 (89) µm, and vessel diameter was 7460 (1057) µm.

#### Maternal and child diet

The median maternal (*N* = 62) total energy intake during pregnancy was 8254 kJ. The median macronutrient composition of energy intake was 45% carbohydrates, 17% protein, and 37% total fat, of which 14% was from saturated fat. Fibre intake was 29 g/day. The median total energy intake of BABY1000 children (*N* = 39) at 24 months was 4761 kJ and the median macronutrient composition of energy intake was 46% carbohydrates, 17% protein, and 37% total fat, of which 15% was from saturated fat. Fibre intake was 14 g/day. Macronutrient distribution did not align with the recommended guidelines for the Australian population.^27^ Both maternal and child diets exceeded the recommended limits for fat intake (upper limit 35%), especially saturated fats (upper limit 10%).

### Primary analysis: excessive weight gain and aIMT

**Fig. 4:**
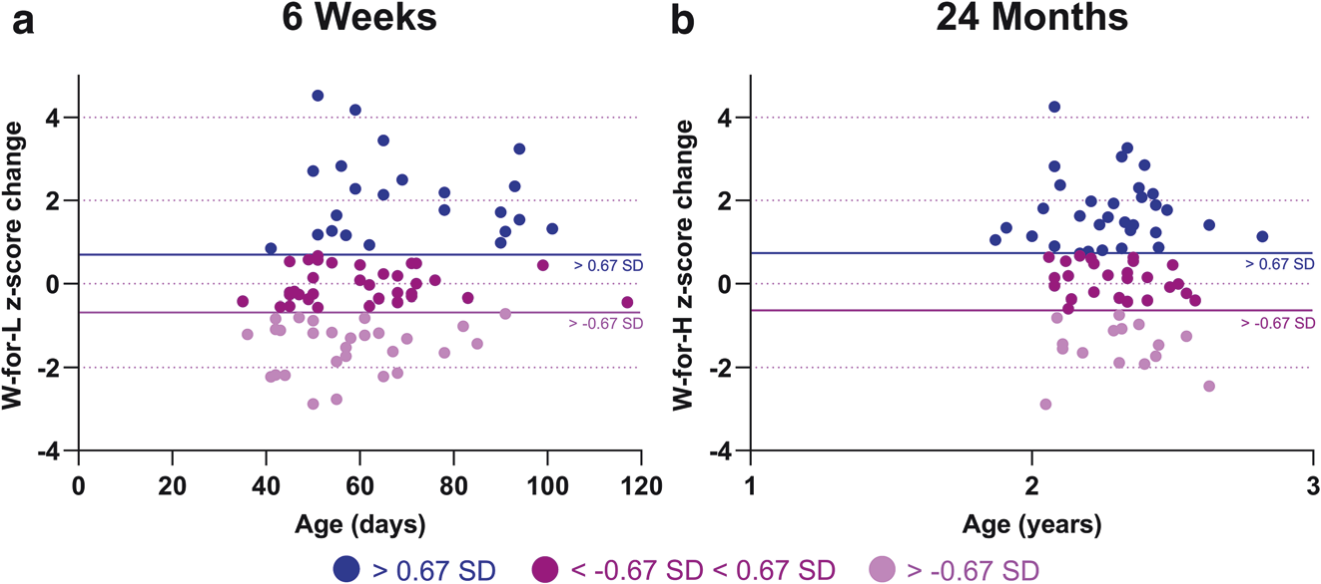
Growth velocity at 6 weeks and 24 months of children in the BABY1000 pilot study. Weight-for-length/height z-scores were calculated using WHO Child Growth Standards. Growth velocity was calculated as the change in z-score from birth to the respective follow-up [(**a**) 6 weeks or (**b**) 24 months]. Reference lines indicate a change in weight-for-length/height z-score of ±0.67 SD. Reference lines indicate a change in weight-for-length/height z-score of ±0.67 SD. Growth velocity data were available from 95 infants at 6 weeks and from 82 children at 24 months. SD standard deviation, W-for-L weight-for-length, W-for-H weight for height.

At 6 weeks, 23 infants (26.4%) were classified as having EWG, 34 (39.1%) as having on-track growth, and 30 (34.5%) as having decelerated growth (Fig. 4). EWG was associated with significantly shorter gestation compared to on-track infants (*p* = 0.02), and with significantly shorter gestation (*p* < 0.001), lower birthweight (*p* < 0.001), and smaller head circumference (*p* = 0.01) compared to those with decelerated growth. No differences were observed in birthweight centile classifications across groups (Supplementary Table S1).

**Fig. 5:**
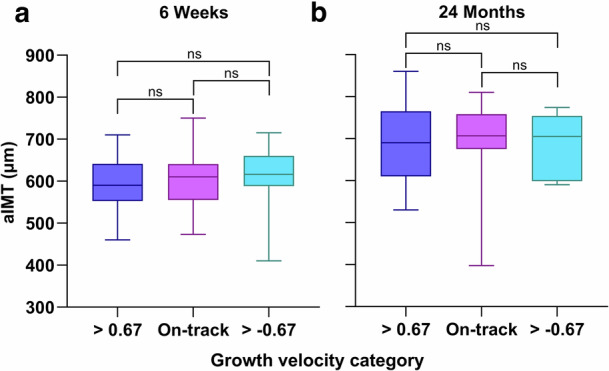
Aortic intima-media thickness by growth velocity category at 6 weeks and 24 months in children from the BABY1000 pilot study. Weight-for-length/height z-scores were calculated using WHO Child Growth Standards. Growth velocity was calculated as the change in z-score from birth to the respective follow-up [(**a**) 6 weeks or (**b**) 24 months]. A change in weight-for length/height z-score greater than ±0.67 SD corresponds to an increase or decrease in two percentiles, respectively. “On-track” growth velocity refers to a change between –0.67 and 0.67. Measurements of both growth velocity and aIMT were available from 69 infants at 6 weeks (<–0.67: On-track: >0.67 = 18:29:22) and from 52 children at 24 months (<–0.67: On-track: >0.67 = 25:19:8). aIMT aortic intima-media thickness, ns not significant, µm micron.

At 24 months, 33 children (45.2%) were classified as having EWG, 25 (34.3%) as having on-track growth, and 15 (20.5%) as having decelerated growth (Fig. 4; Supplementary Table S2). EWG was more common in males (63.6%), although this was not statistically significant. In contrast, decelerated growth was significantly more common in females (73.3%, *p* = 0.02). Children with decelerated growth had significantly lower birthweight (*p* = 0.03), shorter birth length (*p* < 0.001), and smaller head circumference (*p* < 0.01) compared to those with EWG. No differences in birthweight were observed between the EWG and on-track groups.  Those with decelerated growth at 24 months also had significantly lower weight and length at 6 weeks (*p* = 0.03). No differences in early EWG (birth to 6 weeks) were observed across 24-month growth groups (Supplementary Table S2). There were no significant differences in average aIMT across the three growth categories at both timepoints (Fig. 5).

### Secondary exploratory analyses

#### Maternal characteristics and infant outcomes at birth and 6 weeks

Maternal age was significantly associated with lower birthweight (β = –24.8, 95% CI [–39.6, –10.0], *p* = 0.001), while maternal weight (β = 6.2, 95% CI [1.2, 11.2], *p* = 0.02), height (β = 25.2, 95% CI [16.8, 34.0], *p* < 0.001), and gestational weight gain (β = 25.0, 95% CI [8.0, 42.0], *p* = 0.004) were positively associated with birthweight. Primiparity, maternal height, and percentage body fat were associated with lower infant growth velocity at 6 weeks. There were no association between maternal diet with infant birthweight and growth or aIMT at 6 weeks (Supplementary Table S3).

#### Child characteristics and their relationship with EWG and aIMT

At 6 weeks, higher birthweight, birthweight z-score, and weight-for-length z-score were associated with decreased growth velocity (*p* < 0.001; Supplementary Table S4). At 24 months, growth velocity was positively associated with birth length and length z-score, and negatively associated with weight-for-length z-score (*p* < 0.001). Growth velocity at 6 weeks predicted growth velocity at 24 months (β = 0.2, 95% CI [0.0, 0.4], *p* = 0.03; Supplementary Table S5). Exclusive breastfeeding was not associated with aIMT at 6 weeks, and no significant associations were observed between child diet and aIMT at 24 months (Supplementary Table S6). Child diet was also not associated with growth velocity at either timepoint (results not shown). No significant associations were found between child body composition and growth velocity or aIMT at either timepoint.

### Multivariate analysis with aortic intima-media thickness

At 6 weeks, after adjusting for confounders, male sex was significantly associated with a higher aIMT (β = 33.2, 95% CI [1.7, 64.8], *p* = 0.04) (Table 3). No other variables, including change in weight-for-length z-score (β = –5.5, 95% CI [–17.2, 6.3], *p* = 0.36) or birthweight (β = 0.0, 95% CI [–0.0, 0.0], *p* = 0.76), were significantly associated with aIMT at this timepoint. At 24 months, no variables, including change in weight-for-length z-score (β = –3.4, 95% CI [–31.0, 24.3], *p* = 0.81) or male sex (β = 26.2, 95% CI [–27.7, 80.2], *p* = 0.33), were significantly associated with aIMT.

**Table 3:** Multivariable determinants of aortic intima-media thickness in children at the 6 weeks and 24 months follow-ups.

*Model*	aIMT(μm)		
	β	(95% CI)	*p-value*
*6 weeks*			
∆ Weight-for-length Z-score	–5.5	–17.2, 6.3	0.36
Birthweight (g)	0.0	–0.0, 0.0	0.76
Sex (male)	33.2	1.7, 64.8	**0.04**
Maternal weight at study entry (kg)	0.0	–1.1, 1.2	0.94
*24 months*			
∆ Weight-for-length Z-score	–3.4	–31.0, 24.3	0.81
Birthweight (g)	–0.0	–0.1, 0.1	0.86
Birth length (cm)	3.0	–26.1, 32.0	0.84
Sex (male)	26.2	–27.7, 80.2	0.33

∆ Difference in values between birth and each timepoint.

Values are unstandardised β-regression coefficients (95% CI). The results shown are an increase in maximum aortic intima-media thickness per unit increase in the independent variable.

Bold values indicate significance (*p* < 0.05).

*aIMT* aortic intima-media thickness.

## Discussion

In this sub-study, we found limited evidence that EWG during the first two years of life impacts child vascular health as measured by aIMT. We hypothesise that our lack of findings may be partly due to our sample size, which was impacted by typical attrition in longitudinal studies, as well as attrition and missing data secondary to the COVID-19 pandemic. Additionally, our sample was generally healthy, therefore there were fewer babies born SGA, as a proxy for restricted growth, compared to the population average. Consequently, we were unable to fully test our hypothesis.

### Maternal characteristics were associated with infant birthweight and growth velocity, but not aIMT

Approximately half of Australian women have overweight or obesity at the start of pregnancy. High pre-pregnancy weight and BMI is associated with an increased risk of gestational diabetes and foetal macrosomia, both of which are associated with aortic wall thickness in the offspring, albeit in small case-control and cross-sectional studies.^32,33^ Similarly, in two recent systematic reviews and meta-analyses, the authors found some evidence from small, heterogeneous studies that maternal age and smoking were associated with pre-clinical atherosclerosis in both aortic^34^ and carotid vascular beds.^35^ As such, maternal health and wellbeing are of particular interest for the vascular health of the offspring. In this study, we found no associations between maternal determinants and infant aIMT at 6 weeks of age. This aligns with findings by McCloskey et al.^13^ who found that while maternal pre-pregnancy BMI was positively associated with birthweight, no other maternal factors were associated with either infant growth or aIMT at 6 weeks. Similarly, we observed that measures of maternal adiposity were associated with infant size at birth; specifically, higher early pregnancy weight and gestational weight gain were positively associated with birthweight. Birthweight, in turn, was significantly associated with growth velocity from birth to 6 weeks, with infants born heavier showing slower relative growth in early infancy. This carry-over effect of birthweight on growth trajectory underscores the potential to target maternal health, particularly pre-pregnancy or early pregnancy weight, to influence early infant growth patterns. To that end, most pregnancy interventions have focused on limiting gestational weight gain with offspring birthweight as a secondary outcome. The latest systematic review and meta-analysis evaluating this showed fewer small and large babies with dietary interventions during the antenatal period.^36^ In terms of preventing growth restriction, there are presently no known interventions.

Interestingly, we found that maternal age was negatively associated with birthweight. This contrasts with the typical positive association between maternal age and birthweight.^37^ One possible explanation is the unique composition of our cohort: most participants were first-time mothers, and the commonly observed positive association between maternal age and birthweight is often driven by higher parity in older women. Alternatively, the observed negative relationship may reflect an underlying pathological process, as older first-time mothers have a higher risk of placental dysfunction.^34^

### Child growth patterns were not associated with vascular health during early childhood

The prevalence of EWG in full-term children born appropriate for gestational age ranges from 20 to 36%.^35^ In contrast, up to 80% of infants born premature or with low birthweight experience EWG, although in their case, it is referred to as “catch-up growth”.^38^ In our study, the prevalence of EWG during early infancy was 26.4%, consistent with previous reports; however, this prevalence increased significantly to 45.3% by 24 months. The discrepancy between our results and those reported in the literature may be attributed to differences in the metrics used to define EWG. While many studies use a change in z-score >0.67 SD as the threshold, the specific z-score applied, such as weight, BMI, or weight-for-length, varies. Additionally, the timeframes assessed, such as 0–12 months or the first two years, differ between studies. In this study, we used the change in weight-for-length/height z-scores. This method has been used before^5^ and allows us to account for the changes in weight relative to length. However, when calculating EWG using change in weight z-scores, the prevalence was lower at 6 weeks (12.6%) and 24 months (34.5%). The choice of metric may depend on the specific aim of the study, but it does make establishing the prevalence of EWG and comparisons across studies challenging. Given the study’s small sample, we are hesitant to interpret this finding any further.

Due to the limited sample size (as a pilot study) and the overall healthy population, we were unable to specifically explore the impact of excess postnatal growth in those born SGA. Instead, we explored how significant deviations from a child’s growth trajectory impacted vascular health. We did not find any significant differences in aIMT between those with EWG, decelerated growth, or on-track growth at 6 weeks or 24 months. In our adjusted analysis at 6 weeks, male sex was associated with aIMT. However, as males were larger, longer, and gained more weight than females by 6 weeks, this finding likely reflects inherent differences in size for males. Accordingly, at the 24-month follow-up, when males and females were comparable in size, we found no association between male sex and aIMT.

Although studies exploring postnatal growth patterns and CVD risk markers are sparse, existing evidence consistently links EWG in infancy with an adverse cardiovascular profile in later life. This includes increased arterial wall thickness in the aortic and carotid vascular beds,^5,6^ higher blood pressure,^5,7,39^ higher pulse wave velocity,^40^ and impaired endothelial dysfunction.^41^ These biomarkers are associated, at times independently, with an increased risk of future cardiovascular events in adults. EWG in infancy is also linked to the development of childhood obesity and obesity-related risk factors, which may persist into adulthood and predispose individuals to an increased risk for CVD. Recent findings from the Special Turku Coronary Risk Factor Intervention Project, a cohort of 552 individuals followed from early childhood, found that participants with aIMT above the 80th percentile throughout adolescence (ages 11-19) had persistently high BMI from infancy.^42^ These findings support a mechanism by which early excessive growth may contribute to vascular changes through sustained increases in body size. The study also found that aIMT was more strongly associated with traditional CVD risk factors, such as blood pressure and blood lipids, than carotid IMT, reinforcing our rationale for using aIMT as a marker of early vascular remodelling.

### Further granularity is needed to identify the role of maternal and child nutrition on EWG and vascular health

Maternal nutrition plays a crucial role in determining offspring birthweight; however, its influence on infant vascular health is unclear. In a cohort of 201 mother-child dyads, Gale et al.^43^ demonstrated that low energy intake during early and late gestation was associated with greater carotid IMT in children at nine years of age. Additionally, in a sub-study of a randomised controlled trial examining the benefits of a low-glycaemic index diet compared to a high-fibre diet during pregnancy in high-risk women, Kizirian et al.^44^ found that offspring in the low-glycaemic index group had significantly lower aIMT at one year of age, but this difference was partly attributed to differences in birthweight between the groups. Conversely, our group has previously not found any relationship between carbohydrate quality and quantity with aIMT^45^ or cardiac autonomic function,^46^ nor between fatty acids with aIMT^47^ in a comparable cohort. However, we have found that the maternal dietary fatty acids influence epigenetic aging^49^ in the offspring, which could be an indirect mechanism through which maternal diet may influence offspring vascular health and CVD risk. Our study was novel in its attempt to assess child diet in relation to aIMT but we did not find any associations.

In the present study, we did not find any evidence that maternal energy intake and macronutrient distribution affected infant birthweight, growth velocity, or aIMT at 6 weeks. Additionally, no associations were observed between child diet, growth velocity, and aIMT. The lack of observed dietary associations may be attributed to several possibilities: (1) the small sample size inherent to a pilot study, compounded by an even smaller subset with both dietary assessment and aIMT data available, (2) the general non-selected rather than high-risk population, and (3) the use of an FFQ to evaluate diet. In the BABY1000 study, an FFQ was selected to minimise participant burden given the repeated study visits and questionnaires required at each timepoint. Future studies with repeated, prospective measurements of dietary intake are needed to capture detailed nutritional exposures during early life and explore their potential associations with cardiometabolic health.

### Strengths and limitations

We consider our study to have several strengths, the first of which is the longitudinal design and collection of a broad range of maternal and child clinical factors, using best practice and validated methodology. Secondly, we used publicly available validated growth standards to characterise size at birth and growth trajectories. The use of standardised growth references can facilitate cross-study comparisons. Lastly, our cohort is broadly representative of women and babies born at RPAH. However, we must acknowledge that this population is primarily English-speaking, tertiary educated, and more affluent, which affects the generalisability of our findings.

Consistent with many prospective and in-depth cohorts, this pilot study has limitations. Firstly, the BABY1000 pilot aimed to assess the feasibility and acceptability of embedding a longitudinal birth cohort study in routine clinical care and was not powered on clinical outcomes. Despite this, the analyses presented here were pre-planned and aligned with the study’s priority questions, formalised in 2016, to explore the relationship between maternal nutrition during pregnancy and offspring cardiometabolic health and to determine the benefits of postnatal nutrition in at-risk groups for cardiometabolic health.^15^ Secondly, data completeness was significantly impacted by external influences such as the COVID-19 pandemic, public health directives and internal challenges secondary to the pandemic, such as staff efficiency, remote data collection, and a limited study budget. Despite these challenges, there were few differences in those active at baseline and those at follow-up, showing limited bias in our participant retention. Thirdly, not all children who returned for the 24-month follow-up had also attended the 6-week visit, which limited our ability to calculate growth trajectories between the two timepoints. To preserve sample size and maintain statistical power, EWG was defined from birth for each timepoint. While this approach allowed for broader inclusion, it may have obscured timing-specific growth effects during later infancy and early childhood. Additionally, the timing of follow-up visits was influenced by external factors. Ideally, frequent follow-ups, aligned with key growth and developmental milestones, are needed to better characterise growth trajectories during early childhood and their potential relationship with early vascular health. 

Lastly, we used two different machines to image aIMT at the two timepoints and two different neonatologists performed the scans. These changes were due to logistical challenges; the examination rooms in the NICU were prioritised for patients undergoing routine health check-ups, limiting their availability for the 24-month follow-up scans, and the availability of the sonographers was affected by patient care needs. We minimised sources of variance in the aIMT measurement by using edge-detection software, which is shown to have greater inter-rater reliability than manual callipers,^11^ and by having all scans analysed by the same blinded individual with demonstrated reliability. However, future studies should aim to standardise ultrasound equipment and sonographers across timepoints to reduce sources of methodological variance. If the above is not possible, and provided the study is large enough, a sensitivity analysis accounting for the above sources of variance should be done.

## Conclusions

In this population-derived pilot birth cohort study, we did not find any association between EWG in infancy and early childhood and aIMT, a pre-clinical marker of atherosclerosis. There was some evidence of a relationship between birthweight and growth velocity between birth and 6 weeks, suggesting that perinatal factors may shape early post-natal growth. While we aimed to explore the influence of maternal diet during pregnancy and child nutrition on growth and vascular health during early childhood, no significant associations were identified. These null findings may be due to the small dietary sub-sample and the limited precision of dietary assessment using a retrospective FFQ. This exploratory sub-analysis was limited by a small sample size, and findings should be confirmed in larger studies.

## Supplementary information

Supplementary Materials

## Data Availability

The datasets analysed during the current study are not publicly available as the participants or their parent/guardian did not consent to them being publicly available, but they are available from the corresponding author upon reasonable request.
