# Supplementary material for: Maternal and child predictors of vascular health in infancy and early childhood
Source: Pediatr Res. 2025 Oct 20;99(6):2357–66. doi: 10.1038/s41390-025-04260-1 (PMC13433261; doi:10.1038/s41390-025-04260-1)
Supplement: Supplementary file 1 [file 41390_2025_4260_MOESM1_ESM.pdf]

Supplementary Materials

Supplementary Table S1. Birth characteristics between children with excessive weight gain in infancy, on-track growth, and decelerated growth at 6 weeks in the BABY1000 pilot study.

Characteristic	EWG Mean (SD) (N = 23)	On-track Mean (SD) (N = 34)	EWG vs On-track *p-value*	Decelerated Mean (SD) (N = 30)	EWG vs Decelerated *p-value*
Sex, N (%)					
Female	9 (39.1)	17 (50.0)	0.42	13 (43.3)	0.76
Male	14 (60.9)	17 (50.0)		17 (56.7)	
Gestational age (weeks)	38.7 (1.2)	39.5 (1.2)	**0.02**	39.8 (0.9)	**< 0.001**
Birthweight (g)	3211 (500)	3398 (383)	0.12	3697 (337)	**< 0.001**
Length (cm)	50.7 (2.4)	50.6 (1.6)	0.77	51.2 (1.5)	0.39
Head circumference (cm)	34.3 (1.3)	34.6 (1.4)	0.37	35.2 (1.0)	**0.01**
Birthweight centile, N (%)					
AGA	20 (87.0)	30 (88.2)	0.89	27 (90.0)	0.20
SGA	2 (8.7)	2 (5.9)		3 (10.0)	
LGA	1 (4.3)	2 (5.9)		0 (0)	

Values are mean (SD) for continuous variables and n (%) for categorical variables.
Independent sample t-test and chi-squared test were used to compare characteristics between baseline and follow-up for continuous (e.g., birthweight) and categorical (e.g., sex) variables, respectively.
Bold values indicated significant differences between groups ($p < 0.05$).
AGA appropriate for gestational age, *EWG* excessive weight gain, *LGA* large for gestational age, *SGA* small for gestational age.

Supplementary Table S2. Birth and 6-week characteristics between children with excessive weight gain in infancy, on-track growth, and decelerated growth at 24 months in the BABY1000 pilot study.

Characteristic	EWG Mean (SD)	On-track Mean (SD)	EWG vs On-track *p-value*	Decelerated Mean (SD)	EWG vs Decelerated *p-value*
Birth	*(N = 33)*	*(N = 25)*		*(N = 15)*	
Sex, N (%)					
Female	12 (36.4)	12 (48.0)	0.37	11 (73.3)	**0.02**
Male	21 (63.6)	13 (52.0)		4 (26.7)	
Gestational age (weeks)	39.4 (1.5)	39.4 (1.2)	1.00	38.8 (1.1)	0.20
Birthweight (g)	3407 (484)	3475 (359)	0.50	3126 (369)	**0.03**
Length (cm)	51.2 (1.9)	50.3 (1.5)	0.09	48.3 (1.5)	**< 0.001**
Head circumference (cm)	34.6 (1.2)	34.9 (1.0)	0.32	33.3	**< 0.01**
Birthweight centile, N (%)					
AGA	29 (87.9)	22 (88.0)	0.97	14 (93.3)	0.75
SGA	3 (9.1)	1 (8.0)		1 (6.7)	
LGA	1 (3.0)	2 (4.0)		0 (0)	
6-weeks	*(N = 26)*	*(N = 20)*		*(N = 7)*	
Weight (kg)	5.2 (0.8)	5.3 (0.8)	0.65	4.5 (0.5)	**0.03**
Length (cm)	57.9 (2.9)	57.8 (2.7)	0.84	55.4 (2.8)	**0.03**
EWG					
Yes	10 (38.5)	4 (20.0)	0.17	1 (14.3)	0.23
No	16 (61.5)	16 (80.0)		6 (85.7)	

Values are mean (SD) for continuous variables and n (%) for categorical variables.
Independent sample t-test and chi-squared test were used to compare characteristics between baseline and follow-up for continuous (e.g., birthweight) and categorical (e.g., sex) variables, respectively.
Bold values indicated significant differences between groups ($p < 0.05$).
AGA appropriate for gestational age, *EWG* excessive weight gain, *LGA* large for gestational age, *SGA* small for gestational age.

Supplementary Table S3. Results of univariate regression of maternal predictors with infant birthweight, growth velocity, and aortic intima-media thickness at 6 weeks.

Variable	Birthweight (g)[e]			Growth velocity at 6 weeks[f]			aIMT (μm)[g]		
	β	(95% CI)	*p-value*	β	(95% CI)	*p-value*	β	(95% CI)	*p-value*
Age (yrs)	-24.8	-39.6, -10.0	**0.001**	0.1	-0.0, 0.2	0.10	0.1	-2.8, 2.9	0.97
Parity	-82.7	-217.2, 51.8	0.23	-0.7	-1.4, -0.0	**0.04**	-18.1	-45.2, 9.0	0.19
Weight (kg)	6.2	1.2, 11.2	**0.02**	-0.0	-0.0, 0.0	0.96	0.0	-0.9, 1.0	0.97
Height (cm	25.4	16.8, 34.0	**< 0.001**	-0.1	-0.1, -0.0	**0.04**	1.5	-0.3, 3.2	0.10
BMI (kg/m^2)	1.2	-14.2, 16.6	0.88	0.0	-0.0, 0.1	0.46	-1.1	-4.0, 1.8	0.44
Fat mass (%)[a]	4.4	-11.5, 20.3	0.58	-0.0	-0.2, -0.0	**0.02**	-1.0	-6.9, 4.8	0.72
Gestational weight gain (kg)[b]	25.0	8.0, 42.0	**0.004**	-0.0	-0.1, 0.0	0.27	0.5	-2.9, 3.9	0.77
Gestational fat mass gain (kg)	12.0	-20.0, 44.0	0.45	0.0	-0.2, 0.2	0.83	-5.2	-15.6, 5.3	0.31
Diabetes in pregnancy[c]	-52.0	-229.8, 125.7	0.56	-0.2	-1.3, 0.6	0.52	16.5	-18.5, 51.5	0.35
Hypertensive disorders in pregnancy[d]	-258.8	-664.0, 146.3	0.21	0.6	-1.7, 1.9	0.60	-9.0	-81.2, 63.2	0.80
Maternal diet									
Total energy intake (kJ/day)	0.0	-0.0, 0.1	0.20	0.0	0.0, 0.0	0.76	0.0	-0.0, 0.0	0.71
Carbohydrate intake (%)	-6.7	-21.0, 7.6	0.36	0.0	-0.0, 0.0	0.40	-1.5	-4.4, 1.4	0.32
Fat intake (%)	3.2	-14.4, 20.9	0.72	-0.0	-0.1, 0.0	0.63	2.5	-1.1, 6.0	0.17
Protein intake (%)	22.3	-10.2, 54.7	0.18	-0.1	-0.2, 0.1	0.39	0.9	-4.5, 6.2	0.75
Fibre (g/day)	2.9	-8.4, 14.1	0.62	0.0	-0.0, 0.1	0.45	-1.5	-3.5, 0.5	0.14

Values are unstandardised β-regression coefficients (95% CI). The results show an increase in the dependent variable per unit increase in the independent variable.
Bold values indicate significance $p < 0.05$.
[a] N = 44 for mothers with a body composition measurement at study entry.
[b] N = 141 for mothers with a gestational weight gain measurement, calculated as difference in weight between 12 and 36 weeks' gestation.
[c] N = 35. Diabetes in pregnancy was defined as inclusive of both gestational diabetes (diabetes first diagnosed during pregnancy) and pre-existing diabetes.
[d] N = 6. Hypertensive disorders in pregnancy were defined as chronic hypertension, gestational hypertension, pre-eclampsia/eclampsia and chronic hypertension superimposed with pre-eclampsia/eclampsia.
[e] N = 208 for maternal predictors and infant birthweight, N = 106 for maternal diet predictors and infant birthweight.
[f] N = 87 for maternal predictors and infant growth velocity at 6 weeks, N = 65 for maternal diet predictors and infant growth velocity at 6 weeks.
[g] N = 86 for maternal predictors and infant aortic IMT, N = 62 for maternal diet predictors and infant aortic IMT.
aIMT aortic intima-media thickness, *BMI* body mass index.

Supplementary Table S4. Results of univariate regression of infant birth characteristics with growth velocity at 6 weeks and 24 months and aortic intima-media thickness at 6 weeks.

Variable	Growth velocity at 6 weeks			Growth velocity at 24 months			aIMT (μm)		
	β	(95% CI)	*p-value*	β	(95% CI)	*p-value*	β	(95% CI)	*p-value*
Sex (male)	0.1	-0.6, 0.8	0.71	0.6	-0.0, 1.3	0.06	21.9	-5.0, 47.8	0.11
Gestational age (wks)	-0.2	-0.5, 1.5	0.31	0.2	-0.1, 0.4	0.24	2.0	-7.9, 11.9	0.69
Birthweight (g)	-0.0	-0.0, -0.0	**<0.001**	0.0	-0.0, 0.0	0.57	0.0	-0.0, 0.0	0.26
Birthweight z-score	-0.7	-1.0, -0.4	**<0.001**	0.1	-0.3, 0.4	0.78	6.1	-6.4, 18.6	0.34
Length (cm)	-0.0	-0.2, 0.1	0.62	0.21	0.1, 0.4	**<0.001**	2.5	-4.2, 9.1	0.46
Length z-score	-0.1	-0.5, 0.3	0.57	0.6	0.3, 0.9	**<0.001**	3.0	-9.7, 15.7	0.64
Weight-for-length z-score	-0.9	-1.2, -0.6	**<0.001**	-0.8	-1.0, -0.5	**<0.001**	6.0	-8.0, 20.0	0.39
Fat mass (%)[a]	-0.1	-0.2, 0.0	0.20	0.1	-0.2, 0.1	0.33	2.8	-4.5, 10.1	0.44
SGA[b]	1.2	-0.4, 2.7	0.14	0.4	-0.8, 1.6	0.48	13.9	-38.3, 66.1	0.60
LGA[b]	-0.4	-1.8, 0.9	0.52	0.6	-1.4, 2.7	0.53	-7.2	-59.3, 44.7	0.78

Values are unstandardised β-regression coefficients (95% CI). The results show an increase in the dependent variable per unit increase in the independent variable.
Bold values indicate significance ($p < 0.05$).
[a] N = 27 for infants with a body composition measurement at birth.
[b] N = 6 of infants with data follow-up were born either SGA or LGA.
aIMT aortic intima-media thickness, *LGA* large for gestational age; *SGA* small for gestational age.

Supplementary Table S5. Results of univariate regression of child parameters and growth velocity at 24 months.

Variable	24 months		
	β	(95% CI)	*p-value*
Weight	0.28	-0.12, 0.68	0.17
Length	0.09	-0.03, 0.21	0.15
BMI	0.09	-0.10, 0.27	0.35
Δ Weight (kg)	0.38	-0.04, 0.80	0.08
Δ Weight z-score	0.16	-0.16, 0.48	0.16
Δ Length/height (cm)	0.01	-0.14, 0.15	0.91
Δ Length/height z-score	-0.22	-0.52, 0.07	0.14
Δ Weight-for-length z-score	0.23	0.03, 0.4	**0.03**
Δ BMI z-score	0.27	0.03, 0.51	**0.03**

Δ Difference in values between birth and the 6-week timepoint.
Values are unstandardised β-regression coefficients (95% CI). The results shown are an increase in maximum aortic intima-media thickness per unit increase in the independent variable.
Bold values indicate significance ($p < 0.05$).
BMI body mass index.

Supplementary Table S6. Results of univariate regression of child growth and diet with aortic intima-media thickness at 6 weeks and 24 months.

Variable	aIMT (μm)					
	6 weeks			24 months		
	β	(95% CI)	*p-value*	β	(95% CI)	*p-value*
Infant growth						
Δ Weight (kg)	20.9	3.5, 38.4	**0.02**	2.4	-13.5, 18.2	0.77
Δ Weight z-score	2.3	-12.4, 16.9	0.76	-2.3	-26.6, 22.0	0.85
Δ Length/height (cm)	9.1	3.9, 14.2	**<0.001**	1.8	-4.2, 7.7	0.56
Δ Length/height z-score	12.4	-0.3, 25.2	0.60	5.3	-13.7, 24.3	0.58
Δ Weight-for-length z-score	-5.3	-15.2, 4.7	0.30	0.3	-20.1, 20.6	0.98
Δ BMI z-score	-5.6	-17.6, 6.4	0.36	-3.5	-24.8, 17.8	0.74
Infant diet						
Exclusive breastfeeding till 6-weeks[a]	9.1	-18.4, 36.5	0.51			
Total energy intake (kJ/day)[b]				0.0	-0.0, 0.0	0.94
Carbohydrate intake (%)				-0.6	-8.4, 7.2	0.88
Fat intake (%)				2.5	-6.8, 11.9	0.59
Protein intake (%)				-0.4	-14.3, 13.5	0.96
Fibre (g/day)				-4.5	-10.0, 1.00	0.11

Δ Difference in values between birth and each timepoint.

Values are unstandardised β-regression coefficients (95% CI). The results shown are an increase in maximum aortic intima-media thickness per unit increase in the independent variable.

Bold values indicate significance ($p < 0.05$).

[a] N = 43 for infants exclusively breastfed till 6 weeks of age.

[b] N = 39 for infants with dietary data from food frequency questionnaire.

aIMT aortic intima-media thickness, *BMI* body mass index.
